# Power-Law Time Exponent *n* and Time-to-Failure in 4H-SiC MOSFETs: Beyond Fixed Reaction–Diffusion Theory

**DOI:** 10.3390/mi16121351

**Published:** 2025-11-28

**Authors:** Mamta Dhyani, Smriti Singh, Nir Tzhayek, Joseph B. Bernstein

**Affiliations:** Department of Electrical and Electronic Engineering, Ariel University, Ariel 40700, Israel; smriti1410@gmail.com (S.S.); nirtzh@ariel.ac.il (N.T.); josephbe@ariel.ac.il (J.B.B.)

**Keywords:** silicon carbide MOSFETs, threshold voltage instability, power-law, trap physics, negative activation energy, Arrhenius analysis

## Abstract

This work investigates bias-temperature instability (BTI) in 1700 V 4H-SiC MOSFETs under realistic 1 MHz switching conditions with simultaneous gate and drain stress. Threshold-voltage measurements reveal that the degradation does not follow the classical Reaction–Diffusion behavior typically assumed for silicon devices. Instead, the power-law exponent *n* shows a clear increase at the largest negative gate bias (−10 V), indicating a field-driven trap-generation mechanism. Temperature-dependent stress tests further show a negative activation energy (−0.466 eV), consistent with degradation accelerating at lower temperatures due to suppressed detrapping. The results demonstrate that conventional silicon BTI models cannot be directly applied to SiC technologies and that fixed-*n* lifetime extrapolation leads to significant errors. A bias-dependent, field-driven framework for estimating time-to-failure is proposed, offering more accurate and practical reliability prediction for high-power SiC converter applications.

## 1. Introduction

Silicon carbide (SiC) MOSFETs have become increasingly important in contemporary power-conversion systems, primarily because they support high efficiency and power density under demanding operating conditions. In contrast to silicon devices, SiC’s wide bandgap (3.26 eV) enables substantially higher breakdown fields, improved thermal performance, and reliable operation at elevated temperatures, with reduced switching losses and enhanced energy efficiency. These material advantages have encouraged their use in high-power applications, where SiC MOSFETs typically outperform silicon-based Insulated-Gate Bipolar Transistor (IGBTs) in terms of on-state resistance and switching speed, potentially reducing system size and cooling requirements by up to 50% [1].

Despite these advantages, SiC MOSFETs face significant reliability challenges that hinder their widespread large-scale deployment. One of the primary concerns is threshold voltage (Vth) instability, exacerbated by bias-temperature instability (BTI) effects, which induce shifts in device parameters under prolonged gate voltage stress. BTI in SiC devices is influenced by the high density of interface traps at the SiC/SiO_2_ boundary, leading to charge trapping and detrapping mechanisms that cause recoverable or permanent Vth shifts, increased on-resistance, and reduced device lifetime. Negative bias temperature instability (NBTI) was first identified in the nineteen-sixties, and since then it has been extensively investigated and modeled as a key limiting factor in MOS device reliability [2]. To this day, there are still many conflicting opinions regarding the physics and even the model used to describe the behavior, even after 40 years of investigation [3]. The reaction–diffusion (R-D) model assumes a fixed-time exponent *n* ≈ 0.2–0.3 as evidence of hydrogen diffusion in silicon [3,4,5]. However, this silicon (Si)-only model cannot be directly applied to SiC, where we observe voltage-dependent *n*, negative activation energy, and field-driven trap dynamics—demanding new reliability frameworks. Our observation of *n* increasing from 0.39 to 0.52 with negative gate bias is consistent with field-accelerated trap dynamics, not diffusion-limited degradation.

This is consistent with Zerarka et al. [6], who showed that the power-law exponent *n* in SiC MOSFETs varies significantly with stress conditions such as gate voltage and temperature, reflecting the activation of deeper oxide traps under stronger fields, and that assuming a constant *n* can lead to inaccurate lifetime predictions under varying stress. In line with their findings, our results also point to a field-dominated, voltage-sensitive BTI mechanism rather than a simple diffusion-controlled process. The empirical plotting approach of Bernstein [7], which is independent of specific trap-physics assumptions, reveals a voltage-dependent *n* in our SiC devices and thus enables field-based TTF models beyond fixed-exponent assumptions.

Nonetheless, some aspects remain clear; for example, negative gate bias causes a larger threshold voltage shift than positive bias (which seems to have nearly no effect on threshold voltage shift). Studies have highlighted similarities and differences in BTI behavior between SiC and silicon MOSFETs, with SiC exhibiting unique temperature dependencies, including low activation energies (~0.08 eV) for certain trapping processes [8,9,10].

## 2. Theoretical Background

We studied the Vth shift because it is the most sensitive measurable electrical indicator of device degradation under BTI, enabling empirical power-law extrapolation of TTF with zero-curvature fitting. By monitoring the Vth, the temperature-dependence with a systematic tracking of the gate-voltage dependence of time exponent *n*, we reveal field-driven degradation kinetics—independent of any assumed trap physics. Our observations challenge the conventional R-D assumptions and highlight the need for bias-aware reliability models in high-power SiC devices.

Temperature has an important impact on the performance and reliability of SiC MOSFETs. Elevated temperatures can cause a rise in ON resistance, which results in larger conduction losses [11]. Several studies on commercial SiC power MOSFETs have reported significant changes in device properties at temperatures above 125 °C and at even higher temperatures, including reduced operating capability and accelerated degradation mechanisms [11,12,13]. These observations underline the importance of understanding temperature-dependent behavior in SiC power devices. Activation energies are often used to assess the temperature impact on BTI, a degradation mechanism in MOSFETs causing parameter shifts under gate stress [6,14]. The temporal variation in the Vth conforms consistently to a power-law model, expressed as:

(1) $$V_{th}(t)=V_{th,0}+A\;t^{n}$$

where *A* represents a constant influenced by factors such as oxide thickness, electric field strength, and temperature, *t* is stress time, and *n* is the time exponent [15], and *V_th_*_,0_ is the initial threshold voltage.

BTI experiments are widely performed to uncover the underlying degradation mechanisms in SiC technology [16,17]. However, these tests primarily assess the impact of gate bias, overlooking the influence of drain bias. It is critical to analyze the evolution of Vth under realistic operating conditions to fully understand the damage caused by repeated switching stress [18] when both gate and drain potentials are considered. This requires designing test circuits that replicate typical switching scenarios, including appropriate frequency, voltage, and duty cycle, while simultaneously measuring key parameters (e.g., Vth) without disrupting device performance. A rapid stress-measurement technique is essential to handle switching frequencies in the MHz range and minimize the interval between stress application and data readout [19,20], preventing misinterpretation due to parameter drift. Conventional parameter analyzers, particularly for packaged devices with significant parasitic capacitances [21], struggle to meet these demands. Therefore, we developed a specialized system for evaluating the stability and performance of cutting-edge commercial SiC devices. To make this distinction explicit, it is useful to compare our setup with conventional BTI measurement systems. High-power SiC MOSFETs are commonly operated at hundreds of kHz to MHz switching frequencies in practical converters [21]. Traditional BTI measurements using parameter analyzers interrupt the stress and use relatively slow measurement loops, with non-negligible delays between stress removal and Vth readout that allow partial recovery and underestimate fast transient trapping in SiC MOSFETs [16,17,20]. Moreover, they are generally limited to static gate-bias stress and cannot maintain simultaneous gate and drain bias or reproduce realistic dynamic switching conditions, for which dedicated switching test circuits are required [18,19]. When it comes to predicting device lifetimes, Power-law-based extrapolation is widely used in industry, offering reliable and insightful predictions that professionals trust [22,23]. It is generally agreed that the shift behavior with time goes with a power law, *t^n^*, where *n* is generally a fraction. We define *n* as the time exponent in $V_{th}\propto t^{n}$. As many authors suggest, there are difficulties with the popular “R-D” model. One such issue is this power-law behavior, which should produce a linear relationship with time. There have been many suggestions and theories relating to diffusion or double diffusion to explain the *n*. We suggest, here, a purely empirical theory for charge trapping that is not dependent on a fixed ‘*n*’, but rather a theoretical construct that allows for ‘*n*’ that can change based on the creation and occupation of traps.

This study provides new insight into BTI behavior in 1700 V SiC MOSFETs under realistic MHz-level switching. Unlike earlier work that relies on static gate stress or silicon-based models, our results show that the power-law exponent *n* varies strongly with gate bias and that the activation energy is negative, indicating a field-driven degradation process. The use of simultaneous gate and drain stress at 1 MHz, combined with bias-dependent *n* extraction, enables a more accurate and practical approach for lifetime prediction in high-power SiC converters.

The paper continues as follows: Section 3 details the measurement setup. Section 4 showcases the compelling data that has been collected from the tested SiC MOSFETs, focusing on the Vth shift and its temperature dependence through activation energy analysis. Specifically, we investigated the impact of negative bias conditions, showing how negative gate bias strongly affects the lifetime of the devices operating at high voltages. Finally, Section 5 offers the conclusions of the paper.

## 3. Experimental Setup

**Figure 1:**
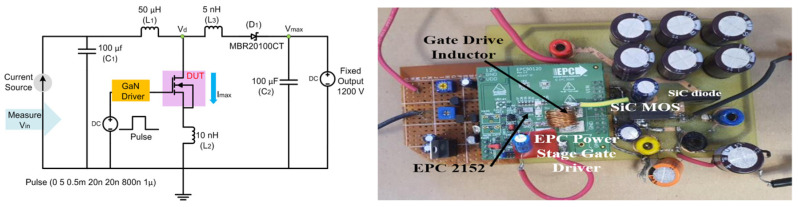
Schematic and Laboratory test setup of SiC-based boost converter, operating in discontinuous mode with soft-switching, designed for efficient power conversion in high-voltage applications.

A simplified diagram of the custom measurement setup is illustrated in Figure 1.

The circuit operates as a SiC power device from Wolfspeed (CREEC2M1000170D) (Wolfspeed, Inc., Durham, NC, USA), rated to 1700 V, which was operated in a boost converter in a switched-mode configuration as shown in Figure 1. A total of five devices from the same manufacturing lot were tested, each dedicated to one of the five gate-bias stress conditions. When the high-speed gate driver (EPC 90120 (Efficient Power Conversion Corporation (EPC), El Segundo, CA, USA) Gallium Nitride (GaN)-based) activates the device under test (DUT) (turning it on), the pulse signal allows current to flow through inductor L_1_, storing energy in its magnetic field while the diode D_1_ remains reverse-biased, isolating the output. Although the EPC90120 incorporates a GaN-based gate-driver stage, the power device evaluated throughout this study is exclusively a commercial SiC MOSFET. No GaN HEMTs were tested or characterized. The GaN technology is used only in the driver circuitry and is not part of the DUT. During the off-state, induced by the negative voltage phases (0 V to −10 V as per setup), the stored energy in L_1_ is released, forward-biasing D_1_ and transferring charge to capacitor C_2_, thereby stepping up the voltage to 1200 V (we used a fixed voltage source) at V_out_. The 70% duty cycle and 1 MHz frequency ensure rapid switching, while soft-switching conditions (zero-current transitions) minimize losses. A square-wave pulse-width modulation (PWM) signal, generated by a pulse generator, drives the GaN-based EPC gate driver. Applying this method to commercially available DUTs enabled us to validate the setup’s effectiveness and offer a qualitative explanation of the physical mechanisms driving the observed Vth drift. Notably, positive Vth drifts (i.e., increases) were detected under our dynamic switching stress conditions. This indicates a net buildup of positive charge in the gate stack (e.g., at or near the SiC/SiO_2_ interface), but a detailed separation of interface and oxide-trap contributions is beyond the scope of this work. BTI-induced threshold-voltage instabilities in 4H-SiC MOSFETs, including the role of interface and near-interfacial oxide traps under positive and negative gate bias, have been extensively studied in the literature [6,9,10,14]. Our dynamic switching results extend these studies to MHz operation with simultaneous gate and drain stress. Capacitors C_1_ and C_2_ stabilize the input and output voltages, respectively, and the current source (I) and voltage monitor (V_1_) enable real-time measurement of V_in_ and Vth shifts, providing insights into the DUT’s behavior under stress.

**Figure 2:**
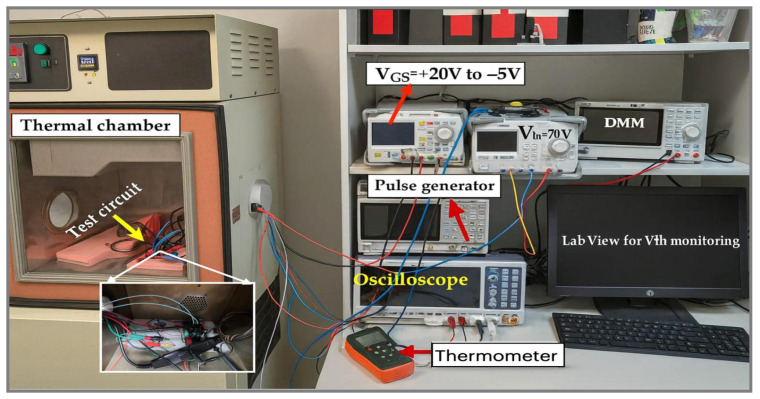
Thermal-chamber BTI test setup. The SiC boost-converter board with GaN gate driver is mounted inside the chamber (**left**), while external supplies, pulse generator, oscilloscope, and LabVIEW PC provide biasing and Vth monitoring, with a thermocouple near the DUT tracking device temperature. Red arrows indicate the locations of the gate-bias supply, pulse generator, oscilloscope, and thermometer in the setup.

For the temperature-dependent BTI measurements, the SiC boost-converter board containing the DUT and the GaN-based gate-driver stage was mounted on an insulated holder inside a programmable thermal chamber (Figure 2). The chamber temperature was set to 5 °C, 25 °C, or 50 °C and controlled by its internal PID regulation, while all external instruments—DC power supply providing V_gs_ from +20 V to −5 V, 70 V input supply to the boost converter, 1 MHz frequency with 70% duty-cycle pulse generator, oscilloscope, digital multimeter, and PC running LabVIEW for Vth monitoring—remained outside the chamber and were connected through electrical feedthroughs. A thermocouple placed next to the SiC MOSFET package was monitored by a thermometer to estimate the device temperature, ensure that it remained within the datasheet limits, and verify that thermal conditions were stable at each chamber setpoint before and during the dynamic switching stress.

It is important to note that this study does not employ RF stepwise power testing in the 41–44 dBm range, which is typically used for GaN HEMT RF reliability evaluation. Instead, the 1700 V SiC MOSFETs are subjected to dynamic switching stress at 1 MHz in a boost converter configuration, and all results are based on the extracted Vth shifts under these switching conditions.

Traditional reliability extrapolation often normalizes degradation (Vth/S_0_) and assumes a fixed-time exponent, leading to optimistic or pessimistic TTF predictions by orders of magnitude due to uncertainty in S_0_. Here, S_0_ is the initial pre-stress Vth at *t* = 0. To avoid this, we plot raw Vth shift (not normalized) versus *t* and optimize *n* such that the second-order coefficient in a quadratic fit is zero—ensuring a perfectly linear relationship [7]. We employed a least-squares-based optimization approach to identify the value of *n* that most effectively aligns with the data, ensuring the best possible fit for our analysis. The time to fail (TTF) is calculated by extrapolating the power-law (−5 V, −7.5 V, and −10 V) and Log-law (0 V and −2.5 V) to a 1 V change in Vth.

## 4. Results and Discussion

Five SiC MOSFETs from the same manufacturing lot were tested, with each device assigned to one specific gate-bias stress condition (0 V, −2.5 V, −5 V, −7.5 V, or −10 V). Thus, the Vth–time characteristics shown for each bias level correspond to the individual device dedicated to that stress condition. Because each bias was applied to a separate device, no averaging or statistical merging of curves was performed; instead, the results directly reflect the degradation behavior representative of each stress level.

For each stress condition, the threshold voltage $V_{\mathrm{th}}(t)$ was monitored as a function of stress time, starting from its fresh value $V_{\mathrm{th},0}$ measured before stress. The time to fail (TTF) was defined as the extrapolated stress time at which the threshold voltage has shifted by 1 V from its initial value, i.e.,

(2) $$V_{\mathrm{th}}(t)-V_{\mathrm{th},0}=1\;V$$

In the weak-stress regime (0 V and −2.5 V), the measured $V_{\mathrm{th}}(t)$ curves were fitted with a logarithmic law,

(3) $$V_{th}\left(t\right)=V_{th,0}+B\ln\left(\frac{t}{t_{0}}\right)$$

and the corresponding TTF was obtained by setting $V_{\mathrm{th}}(t)-V_{\mathrm{th},0}=1\;V$, which gives,

(4) $$\begin{array}{l} 1\;V=B\ln\left(\frac{\mathrm{TTF}}{t_{0}}\right)\;\mathrm{and}\;\mathrm{therefore},\\ \mathrm{TTF}=t_{0}\exp\left(\frac{1\;V}{B}\right) \end{array}$$

For stronger negative gate bias (−5 V, −7.5 V, and −10 V), the degradation follows a power-law dependence,

(5) $$V_{\mathrm{th}}(t)=V_{\mathrm{th},0}+At^{n}$$

with A and n extracted from least-squares fits on a log–log scale; in this case, TTF is obtained from $V_{\mathrm{th}}(t)-V_{\mathrm{th},0}=1\;V=A\;{\left(\mathrm{TTF}\right)}^{n}$, yielding

(6) $$\mathrm{TTF}={\left(\frac{1V}{A}\right)}^{1/n}$$

All TTF values reported, were calculated using this procedure, ensuring a consistent lifetime definition across all stress conditions.

**Figure 3:**
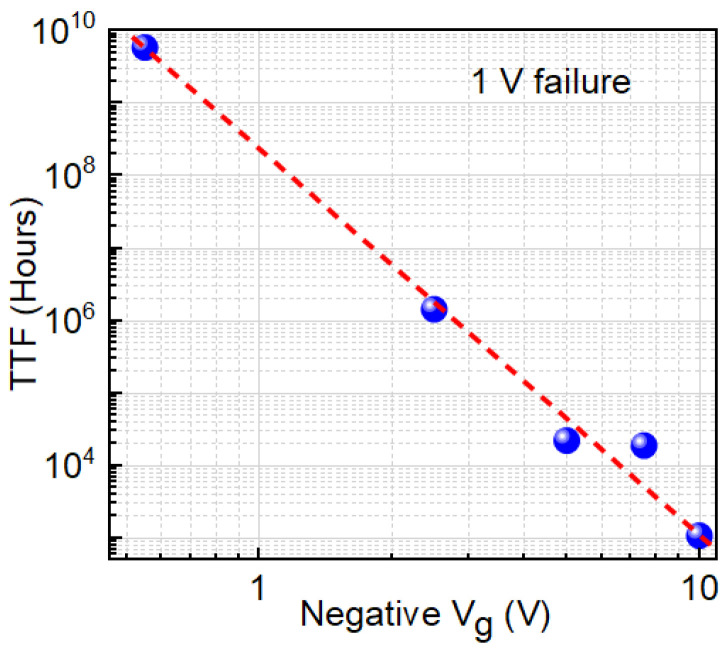
TTF, extrapolated to Vth = 1 V, vs. negative gate bias (−V_g_). TTF decreases with increasing negative V_g_ following a power-law trend, showing that higher field accelerates failure—critical for reliability under high-voltage stress. Blue circles indicate the measured data points; the red dashed line shows the fitted trend.

**Figure 4:**
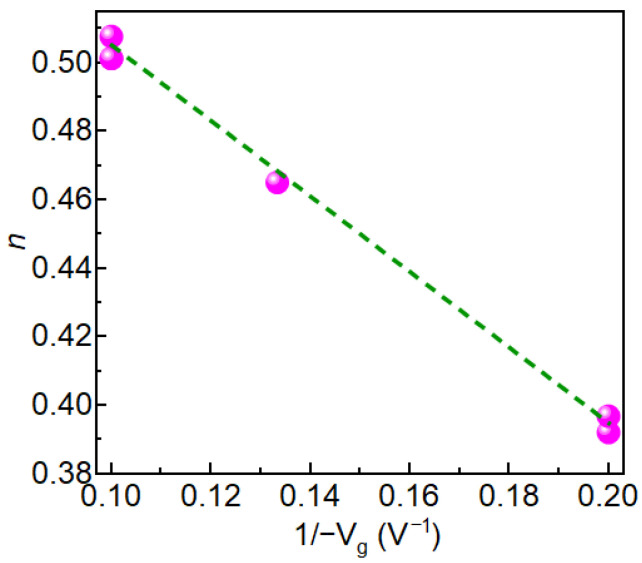
Power-law exponent *n* vs. 1/(−V_g_) for negative gate biases from −5 V to −10 V. *n* increases linearly with increasing 1/(−V_g_), indicating faster degradation kinetics under stronger electric field—consistent with bias adaptive trap dynamics in SiC MOSFETs.Pink circles indicate the measured data points; the green dashed line shows the fitted trend.

**Figure 5:**
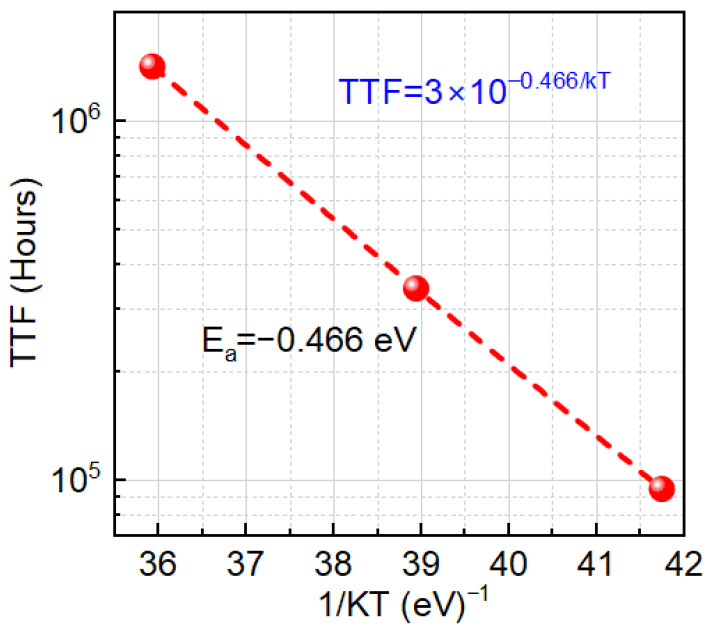
Arrhenius plot of TTF vs. 1/(kT) at −5 V across 5 °C, 25 °C, and 50 °C. TTF increases with increasing temperature, corresponding to an apparent negative activation energy of approximately −0.466 eV.Red circles indicate the measured data points; the red dashed line shows the fitted trend.

**Figure 6:**
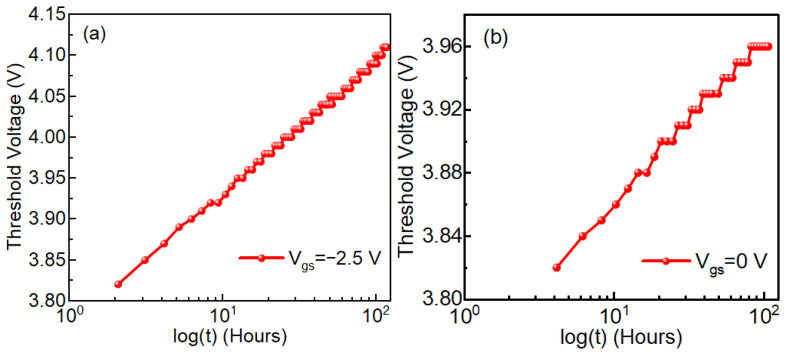
(a) Threshold voltage $V_{\mathrm{th}}$ versus time on a log(t) scale for gate bias $V_{\mathrm{GS}}=-2.5\;V$, showing a clear logarithmic time dependence $\left(V_{\mathrm{th}}\propto log(t)\right)$ and no power-law behaviour (n=0). (b) Threshold voltage $V_{\mathrm{th}}$ versus time on a log(t) scale for gate bias $V_{\mathrm{GS}}=0\;V$, likewise exhibiting logarithmic degradation with n=0. In both cases, the drift is dominated by filling of pre-existing traps, with no significant evidence of new trap generation under these stress conditions.

**Figure 7:**
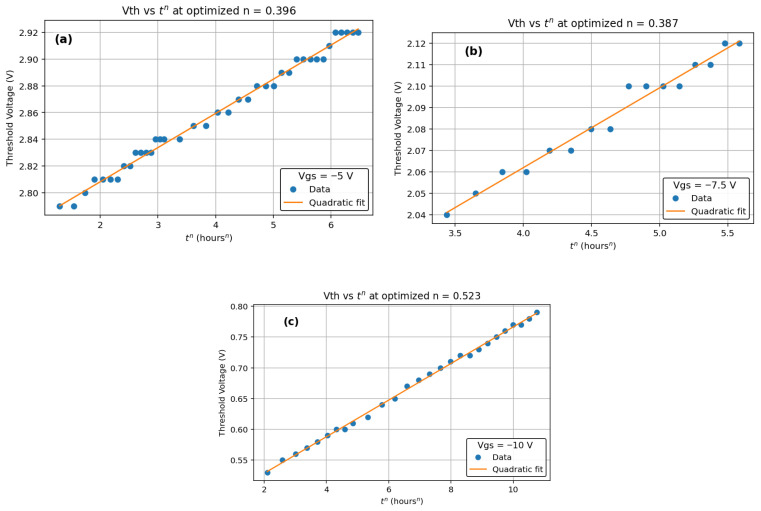
Vth vs. *t^n^* for (**a**) −5 V, (**b**) −7.5 V, and (**c**) −10 V gate bias. All figures clear power-law behavior (Vth ∝ *t^n^*) with *n* > 0 and no saturation over the measured time. The fitted time exponents are *n* = 0.39 (−5 V), *n* = 0.38 (−7.5 V), and *n* = 0.52 (−10 V). The higher value of *n* at the largest negative gate bias (−10 V) suggests that the degradation kinetics are accelerated under stronger oxide field, consistent with field-driven trap generation and non-diffusive, voltage driven BTI behavior in SiC MOSFETs.

**Figure 8:**
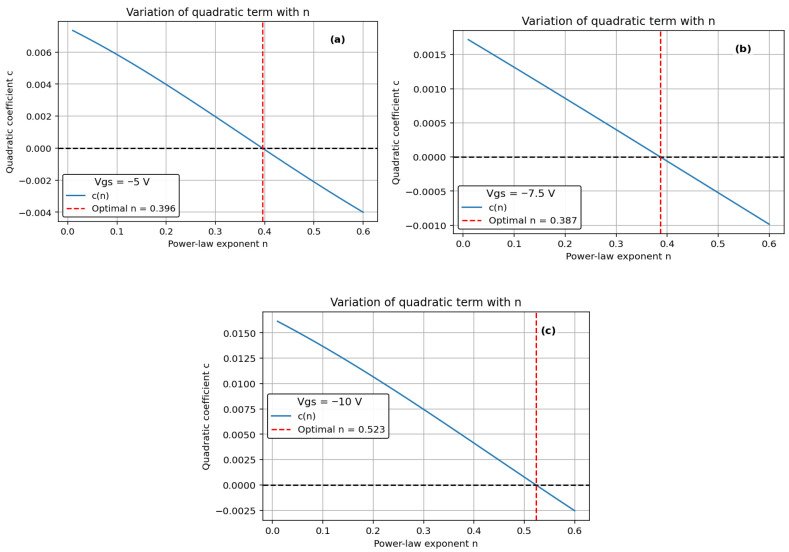
Optimized *n* (Vth ∝ *t^n^* via least square based curve fitting for (**a**) −5 V, (**b**) −7.5 V, (**c**) −10 V. Best-fit *n* = 0.39, *n* = 0.38 and *n* = 0.52. The value of *n* is selected to minimize the second-order term in a polynomial fit (set to zero), ensuring a perfectly linear relationship and accurate TTF extrapolation [16].

We observed TTF vs. −Vg follows a power-law trend showing a decrease in TTF with an increase in negative gate bias (Figure 3). Higher negative gate bias (−V_g_) causes faster degradation, so the device fails sooner—TTF drops. The power-law exponent *n*, derived from Vth vs. *t^n^* plots, increases linearly with 1/(−V_g_) (Figure 4), indicating a bias-adaptive degradation mechanism. The extracted negative activation energy (≈−0.466 eV) in Figure 5 indicates that degradation is more severe at lower temperatures. This behavior is discussed in detail below. Similar behavior has been reported in 4H-SiC MOSFETs and is often interpreted as field-enhanced trapping and detrapping processes rather than diffusion-limited hydrogen motion [6,9,10,14]. While our measurements do not resolve the microscopic trap species directly, the observed negative activation energy is consistent with these field-driven BTI mechanisms. For milder biases (0 V and −2.5 V), *n* = 0, and degradation is logarithmic (Figure 6a,b), indicating no trap creation, whereas for V_g_ ≤ −5 V, power-law fits are excellent (Figure 7a–c), and *n* is extracted accordingly (Figure 8a–c).

We measured Vth under different stress conditions using a LabVIEW-based system, at various temperatures in a thermal chamber. We showed the evolution in threshold voltage versus time to the power of n (for 0 V, −2.5 V, −5 V, −7.5 V, and −10 V) for which the second-order polynomial fit is shown in Figure 6a,b and Figure 7a–c. 

**Figure 9:**
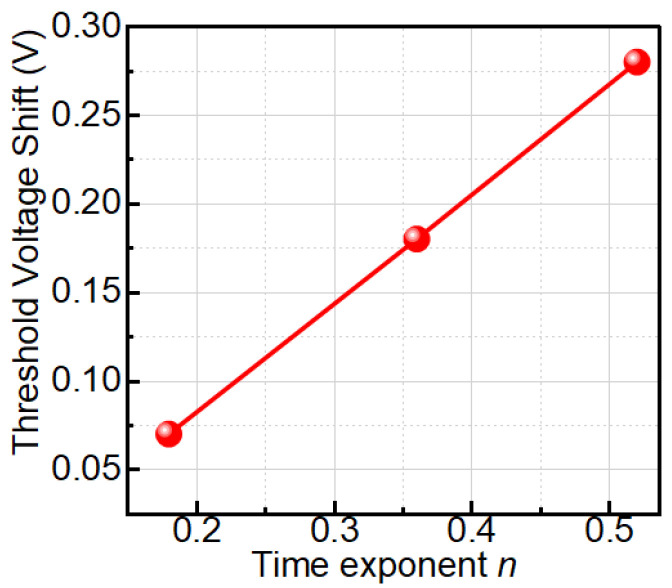
The analysis indicates a correlation between the threshold voltage shift (Vth) and the time exponent (*n).* More negative gate voltages (V_g_) result in more significant Vth shifts, especially at elevated *n* values.

Voltage-dependent values include *n* = 0.39 at −5 V, *n* = 0.38 at −7.5 V, and *n* = 0.52 at −10 V. This study reveals that Vth instability in 1700 V SiC MOSFETs under BTI is field- and temperature-driven. Supported by Arrhenius and logarithmic analysis, these results demonstrate that the power-law exponent *n* in SiC strongly depends on the applied gate bias. As demonstrated in Figure 9, more negative V_g_ leads to larger Vth shifts and higher effective *n* values, unlike silicon R–D theory where *n* ≈ 1/6 remains fixed. Our work highlights the need for field-based models to ensure accurate TTF prediction in high-power SiC devices.

At weak stress (0 V and −2.5 V), the Vth shift follows a logarithmic dependence on time, which we model as Vth ∝ log(t). This behavior is typically associated with the filling and emptying of a broad distribution of pre-existing interface and near-interfacial oxide traps, without significant creation of new defects. Similar logarithmic time dependence and occupancy-dominated behavior have been extensively discussed for BTI in Si/SiO_2_ gate stacks [24,25], where early-time degradation is attributed to the filling and emptying of pre-existing interface and oxide traps. Early-time logarithmic or near-logarithmic Vth drifts have also been reported for 4H-SiC MOSFETs under BTI stress [6,9,10,14,26], supporting the use of this framework for SiC power devices. In this regime, the total number of active traps does not grow with stress time; only their occupancy changes, leading to a slow, sub-linear and near-logarithmic drift that appears as *n* = 0 in our framework (Figure 6a,b). When the negative gate bias exceeds approximately −5 V, the electric field across the gate oxide becomes sufficiently strong to activate field-assisted defect generation processes (such as bond breaking or the creation of new oxide/interface trap sites) [6,9,10,14,25]. In this case, the density of electrically active traps increases with time, and the resulting Vth(t) is better described by a power law, Vth ∝ *t^n^*, with *n* > 0 (Figure 7a–c). The observed “transition” from logarithmic to power-law behavior is therefore not a sudden phase change at −5 V, but a crossover from a regime dominated by occupancy of pre-existing traps (weak stress) to a regime where field-driven trap generation contributes significantly to the total degradation (strong stress).

The Arrhenius analysis at −5 V (Figure 5) yields a negative activation energy of approximately −0.466 eV, i.e., the extracted TTF increases with increasing temperature. This counter-intuitive trend has also been reported for SiC MOSFETs under BTI stress by Puschkarsky et al. [27], who demonstrated that such ‘reverse’ temperature dependence can occur when strong field-driven trapping competes with thermally activated de-trapping. At low temperatures, emission from oxide/interface traps is strongly suppressed, so trapped charge accumulates more efficiently, leading to larger Vth and shorter TTF. At higher temperatures, faster de-trapping reduces the net trapped charge during stress, resulting in longer TTF. This trapping/de-trapping competition produces a negative effective activation energy even though the microscopic trap-generation processes remain thermally activated.

In the power-law regime (V_g_ ≤ −5 V), the extracted time exponent n is not constant but varies with gate bias; in particular, its value at −10 V (*n* ≈ 0.52) is clearly larger than at weaker stress (≈0.39 at −5 V). In our interpretation, *n* acts as an effective measure of how quickly the population of active traps grows with time under a given stress condition. At a stronger negative gate bias, the oxide field is higher, which enhances the charging and activation of near-interfacial and oxide traps and leads to larger Vth shifts and faster degradation [6,9,10,14]. As a result, the cumulative trap density increases more rapidly with time, and the degradation curve becomes “steeper” on a log–log scale, reflecting a larger exponent *n*. This bias-adaptive behavior contrasts with the fixed *n* ≈ 1/6 usually assumed in classical reaction–diffusion models for silicon, and supports a field-driven trap-dynamics picture for SiC.

## 5. Conclusions

This work analyzed BTI-induced threshold-voltage degradation in 1700 V 4H-SiC MOSFETs under realistic MHz switching with simultaneous gate and drain stress, using a converter-based test setup capable of stressing the device at 1 MHz inside a thermal chamber. This configuration allowed us to probe Vth instability under operating conditions that are closer to practical power-converter environments than conventional DC bias experiments.

We showed that the Vth shift in these devices does not follow a single fixed-time exponent or the classical reaction–diffusion behavior commonly used for silicon but instead exhibits a clear crossover from logarithmic degradation at weak stress (0 V and −2.5 V) to power-law behavior at V_g_ ≤ −5 V. The extracted time exponent *n* increases with more negative gate bias, indicating that the degradation kinetics are strongly field-dependent rather than diffusion-limited. At low-oxide fields, the Vth drift is dominated by the occupancy of pre-existing interface and near-interfacial oxide traps, which leads to a slow, nearly logarithmic time dependence that can be represented by *n* ≈ 0. At higher fields, field-assisted activation and generation of additional oxide/interface traps become significant, so that the density of electrically active traps grows with time and the degradation follows a power law with *n* > 0. The bias dependence of *n* therefore provides direct evidence of bias-adaptive trap dynamics, where stronger negative gate bias accelerates the build-up of trapped charge and steepens the time dependence of Vth.

The Arrhenius analysis of TTF at −5 V yielded an apparent negative activation energy of approximately −0.466 eV, i.e., the extracted TTF increases with temperature. This is consistent with a competition between field-driven trap activation and thermally activated detrapping at lower temperatures, where emission from oxide/interface traps is suppressed, so trapped charge is retained and the net Vth shift accumulated during stress is larger, leading to shorter apparent lifetimes. At higher temperatures, faster detrapping and partial recovery during and after stress reduce the measured Vth for a given stress time, and a simple Arrhenius extrapolation then results in a negative effective activation energy. Taken together, the bias dependence of *n*, the logarithmic-to-power-law crossover with increasing V_g_, and the negative activation energy compose a coherent physical picture of a field-dominated, trapping- and trap-generation-controlled BTI mechanism in 4H-SiC MOSFETs under dynamic switching conditions.

Although our measurements are purely electrical and do not identify individual defect species, the combined trends clearly underline the limitations of directly applying silicon-based BTI models with fixed-time exponents and positive activation energies to SiC power devices. For accurate lifetime prediction in high-power SiC converter applications, reliability frameworks must explicitly include the gate-oxide field, the bias dependence of the effective time exponent, and the possibility of non-intuitive temperature trends arising from trapping/detrapping dynamics. The present results, therefore, motivate the development of bias- and field-aware BTI models tailored to SiC technologies and highlight the importance of evaluating device stability under realistic switching conditions, rather than only under static DC gate stress.

## Data Availability

The original contributions presented in this study are included in the article. Further inquiries can be directed to the corresponding author.
